# Focusing on OB-OC-MΦ Axis and miR-23a to Explore the Pathogenesis and Treatment Strategy of Osteoporosis

**DOI:** 10.3389/fendo.2022.891313

**Published:** 2022-07-14

**Authors:** Tian-Liang Ma, Peng Zhu, Zhuo-Ran Ke, Jing-Xian Chen, Yi-He Hu, Jie Xie

**Affiliations:** ^1^ Department of Orthopedics, Xiangya Hospital, Central South University, Changsha, China; ^2^ Hunan Engineering Research Center of Biomedical Metal and Ceramic Implants, Xiangya Hospital, Central South University, Changsha, China; ^3^ XiangYa School of Medicine, Central South University, Changsha, China

**Keywords:** miR-23a, osteoporosis, osteoblast (OB), osteoclast (OC), macrophage, cytokine

## Abstract

Osteoporosis is a bone metabolic disorder characterized by decreased bone density and deteriorated microstructure, which increases the risk of fractures. The imbalance between bone formation and bone resorption results in the occurrence and progression of osteoporosis. Osteoblast-mediated bone formation, osteoclast-mediated bone resorption and macrophage-regulated inflammatory response play a central role in the process of bone remodeling, which together maintain the balance of the osteoblast-osteoclast-macrophage (OB-OC-MΦ) axis under physiological conditions. Bone formation and bone resorption disorders caused by the imbalance of OB-OC-MΦ axis contribute to osteoporosis. Many microRNAs are involved in the regulation of OB-OC-MΦ axis homeostasis, with microRNA-23a (miR-23a) being particularly crucial. MiR-23a is highly expressed in the pathological process of osteoporosis, which eventually leads to the occurrence and further progression of osteoporosis by inhibiting osteogenesis, promoting bone resorption and inflammatory polarization of macrophages. This review focuses on the role and mechanism of miR-23a in regulating the OB-OC-MΦ axis to provide new clinical strategies for the prevention and treatment of osteoporosis.

## 1 Introduction

Osteoporosis is becoming increasingly prevalent with the aging of the world’s population (1). Osteoporosis is a metabolic disease characterized by bone loss, bone density reduction, bone fragility, and increased risk of fractures, and the risk increases with age (2). The dynamic balance between bone formation and resorption underlies bone remodeling, and Osteoporosis tends to occur if an imbalance occurs. At present, osteoporosis has a high disability rate, long treatment cycle, and high cost. A large study in Manitoba, Canada suggested that the cost of treatment of osteoporosis-related fractures or incidental fractures worldwide far exceeds that of many other serious chronic diseases (3, 4). Rehabilitation therapy, including pharmacological and physical therapy, is mainly used, but the clinical outcome is poor (5). Studies have indicated that dysregulation of the OB-OC-MΦ axis is involved in the development of osteoporosis. For example, loss of WNT16 function can lead to increased osteoprotegerin (OPG) expression in osteoblasts, thereby inhibiting osteoclastogenesis, leading to reduced cortical bone mass (6). In addition, chronic inflammation occurring in osteoporosis can derive a range of pro-inflammatory factors, which in turn directly or indirectly promote osteoclast activation (7, 8). Activated macrophages can further secrete pro-inflammatory factors, which induce bone loss by activating osteoclastogenesis (9).

MicroRNAs (miRNAs) are non-coding RNA molecules of about 19 - 22 nucleotides in length that play a vital role in life activities such as cell differentiation, proliferation, and apoptosis. In 1993, the first miRNA was identified as an RNA transcribed from Caenorhabditis elegans. In 2000, the first mammalian miRNA let-7 was discovered, and since then, a series of studies on the genome have revealed many miRNAs and other non-coding RNAs (10). MiR-23a, located on chromosome 19 of the human genome, is transcribed as part of the miR-23a ~ 27a ~ 24-2 cluster. According to the 3’ and 5’ counting rules, the Dicer enzyme cleaves pre-miRNAs from the 3 ‘end and 5’ end positions, respectively, to form mature miRNA-3p and mature miRNA-5p and perform the corresponding functions (11). MiR-23a plays a critical role in inhibiting osteogenesis and promoting osteoclast- and macrophage-mediated inflammation, thereby promoting osteoporosis development. Studies have demonstrated that miR-23a-3p increased in SM-EVs and neoplastic mast cell-derived EVs inhibit osteoblast maturation by suppressing the expression of runt-related transcription factor 2 (Runx2), small mothers against decapentaplegic 1 (SMAD1) and SMAD5 (12). In addition, exosomes containing miR-23a-5p derived from osteoclasts can effectively inhibit ostegenic differentiation and promote the development of osteoporosis by inhibiting Runx2 (13). MiR-23a, which is highly expressed in macrophages, can promote M1 polarization and the generation of pro-inflammatory factors. It accelerates bone resorption by directly or indirectly stimulating osteoclast activation, which encourages the development of osteoporosis (9, 14, 15). Therefore, targeted inhibition of miR-23a to regulate bone metabolic homeostasis is a promising way of treating osteoporosis. At present, however, there are few clinical trials for miR-23a, and whether targeting miR-23a can be used as a bone disease treatment has not been determined. Elucidating the relationship between miR-23a and osteoporosis can not only provide additional tools for clinical identification of patients at risk of osteoporosis, but also provide more information for the development of targeted miRNA therapies as potential interventions to prevent bone loss. Therefore, it is vital for further studies of miR-23a in osteoporosis.

## 2 OB-OC-MΦ Axis in Osteoporosis

**Figure 1:**
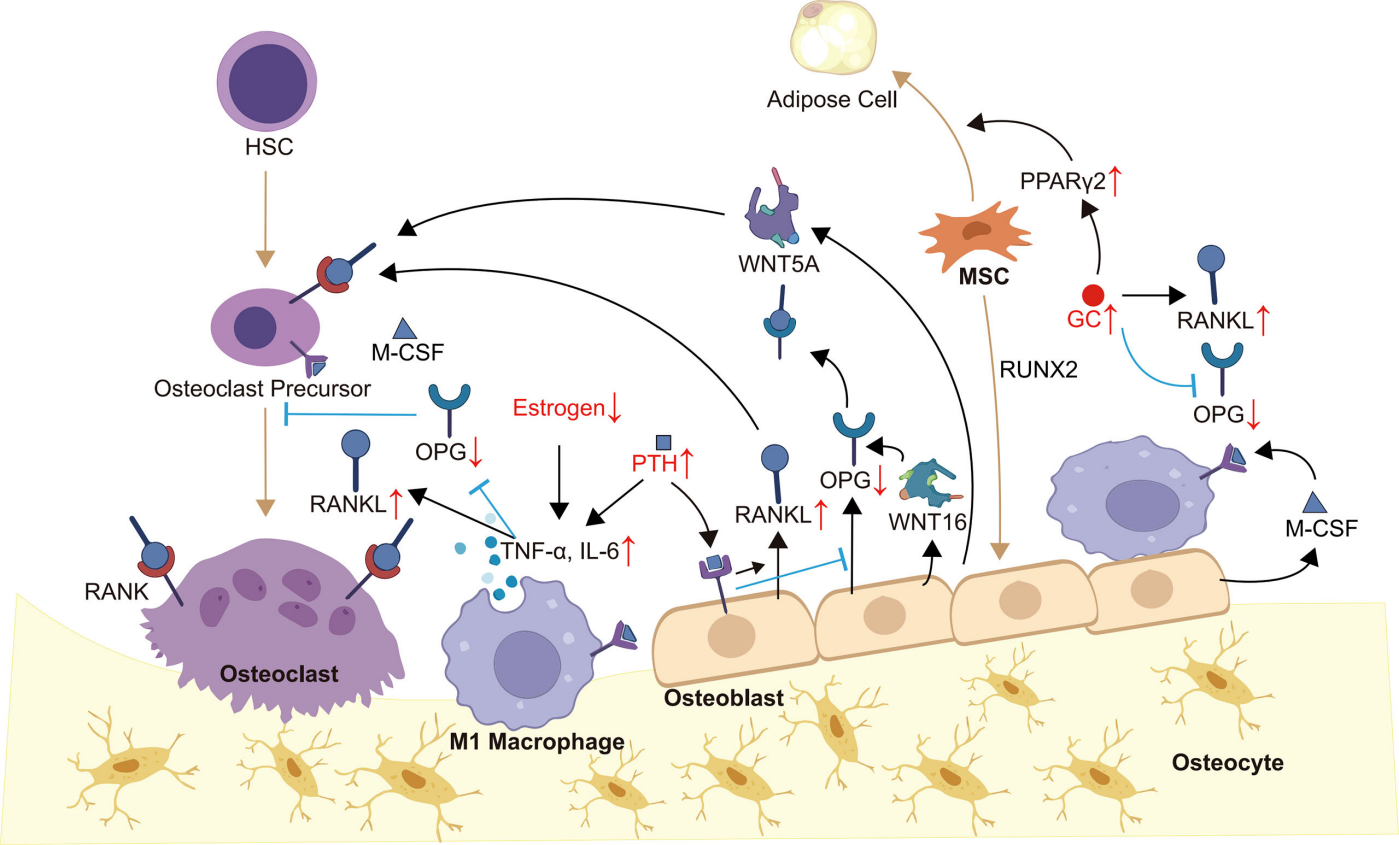
OB-OC-MΦ axis in osteoporosis. Members of the OB-OC-MΦ axis maintain the axis’s equilibrium through intricate communication, which is mostly shown in appropriate levels of cytokines such as RANK, RNAKL, OPG, M-CSF, WNT5A, WNT16, and TNF-α. Endogenous or exogenous factors, such as evident inflammatory responses and aberrant hormone levels, might alter the levels of various cytokines, causing an imbalance of the OB-OC-MΦ axis and, ultimately, the occurrence and progression of osteoporosis. Brown arrows indicate the direction of cell differentiation, and small red arrows indicate abnormal changes in hormone or cytokine levels.

Bone remodeling is a highly coordinated and dynamic process of replacing old or damaged bone, which is necessary to guarantee bone health (16, 17). Under physiological conditions, bone remodeling is regulated by hormones, cytokines and other factors to ensure bone strength and mineral homeostasis (16, 18). OB-OC-MΦ axis is the core of bone remodeling regulation, and its imbalance will usually lead to a variety of bone metabolism diseases, including osteoporosis (**Figure 1**).

### 2.1 Composition

Bone tissue cells include osteoprogenitors, osteoblasts, osteocytes, bone lining cells, and osteoclasts, in which osteoblasts, osteoclasts, macrophages and their precursors constitute the OB-OC-MΦ axis. Osteoblasts located on the surface of bone are developed from mesenchymal stem cells (MSCs) and are characterized by the abundant rough endoplasmic reticulum and Golgi complex (19). Osteoblasts play an essential role in bone formation *via* secreting osteoid (16). Osteoblasts become osteocytes when they are embedded in osteoid, which act as sensors and coordinators of the bone remodeling process (16, 19). Most investigators concur that osteoblasts can recruit osteoclasts and affect their formation and function (15, 20). Mature osteoclasts are multinucleated giant cells derived from hematopoietic stem cells (HSCs) (21, 22), and they can dissolve bone matrix by secreting organic acids and proteolytic enzymes (23). The excessive generation and activation of osteoclasts will lead to pathologic bone loss, which underlies osteoporosis (24). Facts have proved that inflammation is closely related to bone loss (25). Macrophages, known for their phagocytosis, can polarize into two types, M1 and M2, which have pro-inflammatory and anti-inflammatory effects respectively (26, 27). It’s worth mentioning that macrophage polarization can shift between M1 and M2 depending on the local microenvironment (28). Normally, macrophages maintain bone homeostasis in coordination with osteoblasts and osteoclasts by secreting a large number of cytokines (29). For example, M1 macrophages-derived M1 cytokines, including tumor necrosis factor α (TNF-α), IL-1β, IL-6, IL-12 and other pro-inflammatory cytokines, while M2 cytokine library includes IL-4, IL-13, bone morphogenetic protein 2 (BMP-2), transforming growth factor beta (TGF-β), and so on (30). Furthermore, bone resident macrophages such as bone marrow macrophages, osteal macrophages also contribute to bone homeostasis and remodeling.

### 2.2 Crosstalk Molecular Regulatory Mechanisms

Under physiological conditions, there is a complex and perfect communication system between members of the OB-OC-MΦ axis, which together maintain bone homeostasis through crosstalk in various ways. **Table 1** shows the effects of different cell-derived cytokines in the OB-OC-MΦ axis.

**Table 1:** Summary of the effects of cytokines in the OB-OC-MΦ axis.

Cytokine	Source	Action	Reference
M-CSF	OB	Promotes survival, differentiation, cell migration and activity of OC and MΦ	(31, 32)
RANKL	OB	Promotes OC differentiation and activation; inhibits OC apoptosis	(33)
OPG	OB	Inhibits the recruitment and activation of OC; inhibits osteoclastogenesis	(21, 34, 35)
WNT5A	OB	Enhances RANK expression; promotes osteoclastogenesis	(36, 37)
WNT16	OB	Upregulates OPG; inhibits osteoclastogenesis	(36)
WNT10B	OC	Stimulates the recruitment, proliferation and differentiation of OB	(38, 39)
BMP-2	OC, M2 MΦ	Stimulates the recruitment, proliferation, differentiation and activity of OB; regulates the production of RANKL and M-CSF; promotes OC formation and differentiation indirectly	(38, 39)
Semaphorin 4D	OC	Inhibits bone formation; suppresses IGF-1 signaling; modulates OB motility	(40)
TNF-α	M1 MΦ	Promotes OC progenitors differentiation/amount directly; enhances RANKL secretion	(41–43)
IL-1	M1 MΦ	Stimulates RANKL secretion; Downregulates OPG levels; promotes osteoclastogenesis	(44)
IL-6	M1 MΦ	Stimulates RANKL secretion; promotes OC formation and development	(45–47)
IL-12	M1 MΦ	Inhibits the formation activation, and survival of OC	(48, 49)
IL-18	M1 MΦ	Inhibits the formation of OC; synergizes with IL-12	(48, 49)
IL-10	M2 MΦ	Interferes with NFATc1 expression and nuclear translocation; upregulates OPG synthesis; down-regulates RANKL and M-CSF expression; blocks IL-1, IL-6 and TNF-α expression; inhibits OC formation and activity	(50–52)
OPN	M2 MΦ	Improves OC activity; Increases OC attachment	(48, 53)
OSM	M1 MΦ	Activates STAT3 phosphorylation; activates Runx2; inhibits sclerostin expression; targets WNT5A to promote osteoclastogenesis	(54, 55)

#### 2.2.1 Osteoblasts and Osteoclasts

In the process of bone remodeling, osteoblasts and osteoclasts communicate with each other mainly through cell-cell contact and diffusible paracrine factors (56). When osteoblasts and osteoclasts come into contact, gap junctions can be formed, which are beneficial to material and information exchange. Cell-cell contact, in particular, allows bidirectional activation signal transduction *via* membrane-bound mediators, regulating each other’s differentiation and survival (36). In addition, osteoblasts can release factors to regulate osteoclasts survival and differentiation, such as macrophage colony-stimulating factor (M-CSF, also called CSF1), receptor activator of nuclear factor κ-B (RANK) ligand (RANKL), OPG, WNT5A and WNT16 (36). In terms of mechanism, M-CSF can bind to receptors on osteoclasts and macrophages thereby affecting their survival, differentiation, cell migration and activity (31, 32). RANKL and OPG are secreted by osteoblasts, while RANK presents on the surfaces of osteoclast precursors and mature osteoclasts. The combination of RANKL and RANK will promote the differentiation of osteoclast precursors into osteoclasts and inhibit the apoptosis of osteoclasts (33). OPG secreted by osteoblasts is the decoy receptor of RANK, which inhibits the recruitment and activation of osteoclasts by binding to RANKL (34, 35). Osteoblasts also regulate osteoclastogenesis through WNT signaling pathways. For example, WNT5A expressed by osteoblasts enhances RANK expression through receptor tyrosine kinase-like orphan receptor 2, while WNT16 upregulates OPG (36, 37). In turn, WNT10B and BMPs such as BMP2 and BMP6 secreted by osteoclasts stimulate the recruitment, proliferation and differentiation of osteoblasts (38, 39). Besides, osteoclast-derived semaphorin 4D inhibits insulin-like growth factor-1 (IGF-1) signaling and regulates the motility of osteoblasts by binding to receptors on osteoblasts to inhibit bone formation (40).

#### 2.2.2 Macrophages and Osteoclasts

Macrophages and osteoclasts are different products of myeloid progenitors that compete with each other (57). HSCs differentiate into monocytes/macrophages, followed by the proliferation of pre-osteoclasts and maturation (48). At the moment, it is widely assumed that the monocyte/macrophages are not only the precursors of osteoclasts, but also essential regulators of bone homeostasis. As previously stated, macrophages exhibit distinct polarization in different microenvironments, hence their impacts on osteoclasts differ. Cytokines released by M1 and M2 macrophages can both affect the differentiation and activity of osteoclasts (48). For instance, TNF-α can directly increase the number and/or differentiation of osteoclast progenitor cells, or indirectly promote osteoclast formation by enhancing the secretion of RANKL by osteoblasts and other cells (41–43). IL-1 can stimulate osteoclastogenesis by decreasing OPG levels and increasing RANKL levels (44). IL-6 not only has the ability to stimulate RANKL secretion, but also can mediate the action of TNF-α, and IL-1, and activate JAK/STAT3 (Janus kinase/Signal transducer and activator of transcription 3) pathway to promote osteoclast formation and development (45–47). It cannot be ignored that cytokines produced by M1 macrophages also have negative effects on osteoclasts, for example, IL-12 and IL-18 can inhibit the formation of osteoclasts (48, 49). M2 macrophages can secrete anti-inflammatory factors and bone growth factors, among which IL-10, BMP-2 and osteopontin (OPN) are typical. Studies have proved that IL-10 can inhibit the formation of osteoclasts (58). In mechanism, IL-10 can interfere with NFATc1 expression and nuclear translocation (50), upregulate OPG synthesis, down-regulate RANKL and M-CSF expression (51), and block the expression of pro-osteoclast factors such as IL-1, IL-6 and TNF-α (52) to inhibit osteoclast formation and activity. While BMP-2 promotes the formation, differentiation and activity of osteoclasts by enhancing the synthesis and secretion of RANKL and M-CSF (48, 59). OPN can also improve the activity of osteoclasts and help them adhere to the bone surface (48, 53).

#### 2.2.3 Macrophages, MSCs and Osteoblasts

Resident macrophages and inflammatory macrophages can communicate with MSCs and osteoblasts, which is essential for bone formation. Osteal macrophages, for example, have direct contact with osteoblasts, which can promote bone matrix secretion and mineralization (60). The communication between macrophages and osteoblasts *via* cytokines was discussed in the preceding section and the relationship between macrophages and MSCs will be emphasized here. It has been proved that early inflammation and the recruitment of monocyte macrophages are required for effective repair (61). The direct contact between M1 macrophages and MSCs can produce oncostatin M (OSM), which is dependent on prostaglandin E2 and cyclooxygenase 2 (54, 62). OSM activates STAT3 phosphorylation, which can regulate osteoblast differentiation *via* activating Runx2 and inhibiting the expression of sclerostin, an osteocyte-derived mineralization inhibitor (55). What’s more, OSM signaling through STAT3 can directly target WNT5A to promote the differentiation of MSCs into osteoblast (54). More interestingly, STAT3 signaling can lead to the upregulation of OSM receptors to amplify its effects. In turn, macrophages cocultured with MSCs have higher CD206 expression, higher IL-10 and IL-12p40 production, and lower TNF-α, IL-6 and IL-12p70 production (63, 64). It can be guessed that MSCs induce M1 macrophage phenotype to transform into M2 phenotype to coordinate osteogenesis. In summary, the activation of MSCs by inflammatory mediators leads to the transformation of M1 macrophages into M2 phenotype, while OSM derived from M1 macrophages promotes the differentiation of MSCs into osteoblasts.

### 2.3 Manifestation and Mechanism of OB-OC-MΦ Axis Imbalance in Osteoporosis

The OB-OC-MΦ axis imbalance will result in the onset and progression of osteoporosis, the primary mechanism of which is reduced bone production and increased bone resorption. Osteoporosis pathology is typically accompanied by prominent inflammation and aberrant hormone signaling.

The pathogenesis of osteoporosis is multifactorial, but it is usually accompanied by a local or systemic significant increase of pro-inflammatory cytokines, which further mediates the imbalance of the OB-OC-MΦ axis. For example, the level of TNF-α in patients with osteoporosis is increased. On the one hand, TNF-α induces osteoclastogenesis through the RANK-RANKL-OPG pathway (65). On the other hand, it inhibits osteoblasts by promoting the production of Dickkopf-1 (66). During the development of osteoporosis, the inflammatory cytokine IL-6 is secreted by various cells, such as lymphocytes, macrophages, and osteoblasts. IL-6 initiates signal transduction by forming a complex with the IL-6R and glycoprotein 130 (gp130) (67). In bone metabolism, IL-6 induces the formation and activation of osteoclasts and inhibits their apoptosis, which depends on IL-6R/gp130/RANKL pathway (68–70). Furthermore, other IL-6 cytokine family members, such as IL-11 and cardiotrophin 1, also support osteoclastogenesis (71, 72). To summarize, the inflammatory response in the process of osteoporosis stimulates the osteoclast offset of the OB-OC-MΦ axis, which promotes the disease’s progression.

Aberrant hormone signaling is a major cause of osteoporosis, which can lead to the imbalance of the OB-OC-MΦ axis. It is found that the acceleration of bone loss caused by estrogen deficiency is closely related to the overactivation of the RANK-RANKL pathway, and the incidence rate is higher in menopausal women and people with breast cancer (73, 74). In addition, the occurrence of osteoporosis induced by abnormal estrogen signaling is also related to the amplification of inflammation. Estrogen withdrawal will promote the production of a series of pro-inflammatory cytokines, such as TNF-α, leading to bone loss (75, 76). Parathyroid hormone (PTH) is a crucial regulator of bone development, and patients with hyperparathyroidism are more likely to develop osteoporosis (77, 78). There are PTH receptors on the surface of osteoblasts and osteocytes. PTH can increase the mRNA encoding for RANKL while inhibiting the mRNA encoding for OPG (79), thereby increasing the RANKL/OPG ratio and promoting osteoclast production (45, 80, 81). PTH can also stimulate the production of pro-inflammatory cytokines, such as TNF-α by CD4^+^ T cells (82). In addition, PTH fosters the production of various acids and enzymes related to bone resorption, such as lactic acid, matrix metalloproteinase 9 (MMP9) and MMP13 (83–85). Glucocorticoid (GC) is commonly used to alleviate inflammation (86), however, it also cause osteoporosis (87). GC directly or indirectly inhibits the generation and function of osteoblasts and induces their apoptosis (88, 89), such as upregulating peroxisome proliferator-activated receptor γ2 (PPARγ2) to differentiate MSCs into adipocytes (87). GC can also increase the RANKL/OPG ratio to promote osteoclastogenesis (88, 90). To summarize, the occurrence of osteoporosis is commonly accompanied by aberrant levels of a range of hormones, which facilitates the progression of osteoporosis by mediating the OB-OC-MΦ axis imbalance.

In summary, the members of the OB-OC-MΦ axis interact and restrict each other in various ways to maintain its balance. The primary mechanism of osteoporosis is the OB-OC-MΦ axis imbalance, which is manifested by the impeded bone formation and enhanced bone resorption. The pathological process of osteoporosis patients is usually accompanied by inflammation or abnormal hormone signaling, which could trigger the imbalance of OB-OC-MΦ axis.

## 3 Regulation of OB-OC-MΦ Axis by miR-23a

### 3.1 MiR-23a and Osteoblasts and Osteoclasts

A series of miRNA have been identified to be involved in bone formation and bone resorption (91). For instance, osteoclast-derived exosomal miR-214 can inhibit the activity of osteoblasts by targeting EphrinA2/EphA2 and activating transcription factor 4 (92–94), and plays a key role in osteoclast differentiation *via* autocrine (95). Exosomal miR-503-3p from osteoblasts has been found to decrease RANK production and RANKL-induced osteoclast differentiation (96). The exosomes or microvesicles derived from various cells contain miR-23a, which is closely related to the OB-OC-MΦ axis. Exosomes can be absorbed by cells through autocrine or paracrine pathways, and can also be absorbed by distant target tissues or organs through circulatory system. In addition to exosomes or extracellular vesicles, endogenous or exogenous factors may also cause the expression level of miR-23a in cells to increase. By targeted regulation of related protein-coding genes, miR-23a can disrupt the balance between osteoblast-mediated bone formation and osteoclast-mediated bone erosion and ultimately contributes to osteoporosis.

MiR-23a mainly inhibits osteogenic differentiation by targeting Runx2, WNT signaling pathway and PGC-1α. Runx2, as the most critical downstream molecule of BMP signaling, is the essential transcription factor of osteoblast formation and the critical driving factor of osteogenesis (97, 98). Runx2 and Wnt/β-catenin signaling promote the differentiation of MSCs into immature osteoblasts while suppressing differentiation into chondrocytes or adipocytes (99). It is worth noting that Runx2 inhibits the maturation of osteoblasts and the transformation of osteoblasts into osteoblasts (99). Bioinformatics prediction and double luciferase reporter gene detection demonstrated that miR-23a can directly bind to the 3’UTR of Runx2 (100), thereby inhibiting osteogenesis (101). Studies have found that patients with systemic mastocytosis are often accompanied by pathological features of osteoporosis. The extracellular vesicles released by mast cells contain a large amount of miR-23a-5p, which has an obvious Runx2 inhibitory effect (12). Exosomes generated from osteoclasts contain a high concentration of miR-23a, which inhibits osteogenic differentiation and osteoblast activity (13). In addition, Runx2 and Stabilin 2 (STAB2) synergistically promote osteoblast differentiation (97). Exogenously expressed miR-23a can target and inhibit SATB2 and indirectly inhibit the osteogenic effect of Runx2 (102). MiR-23a can also inhibit WNT/β-catenin signaling pathway (103), because it directly targets low-density lipoprotein-receptor-related protein 5 (LRP5) (104). Activating the WNT/β-catenin signaling pathway can promote the differentiation of bone marrow MSCs into osteoblasts and prevent bone loss (105). As well as inhibiting RUNX2 in immature osteoblasts, miR-23a can inhibit Prdm16 from regulating TGF-β signaling pathway to promote the terminal differentiation of osteoblasts. What’s more, miR-23a in MSCs from irradiation activated gingival fibroblasts can target C-X-C Motif Chemokine Ligand 12 (CXCL12) to inhibit osteogenic differentiation (106, 107). Besides, miR-23a may also inhibit bone formation through the miR-23a-PGC-1α pathway. According to research, miR-23a can bind to the 3’UTR of PGC-1α and limit its expression (108). PGC-1α determines the fate of the stem cell lineage to differentiate into osteoblasts or adipocytes, and the absence of PGC-1α leads to reduced bone formation and indirect promotion of bone resorption (109). In addition, the expression of PGC-1α decreases with aging (109), which may be one of the reasons why osteoporosis is much more common in the elderly. However, there is no direct evidence that miR-23a regulates the differentiation direction of MSCs by inhibiting PGC-1α. MiR-23a from multiple sources can be absorbed by osteoblast precursors and prevent them from differentiating into osteoblasts, or promote the terminal differentiation of osteoblasts, and finally destroying the balance of OB-OC-MΦ axis.

**Figure 2:**
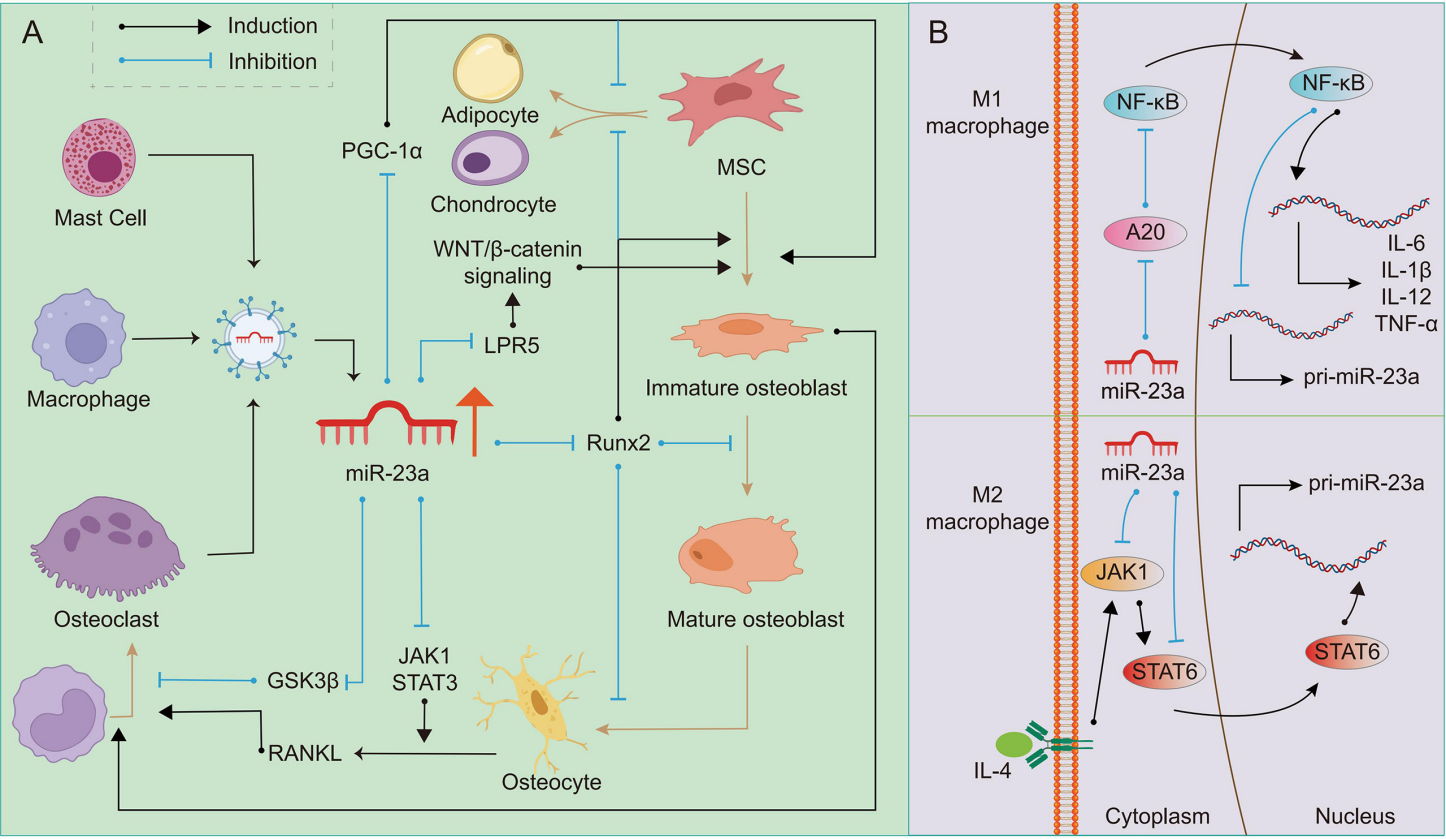
Regulation of miR-23a on OB-OC-MΦ axis. A high level of miR-23a impairs the balance of the OB-OC-MΦ axis, leading to the occurrence of osteoporosis. **(A)** Many kinds of cells can secrete exosomes or extracellular vesicles containing miR-23a. On the one hand, miR-23a can target Runx2, LPR5 and PGC1 to regulate osteoblast differentiation and maturation; On the other hand, it can target GSK3β and JAK1/STAT3 to regulate osteoclast differentiation. The regulation of miR-23a on osteoblasts can also indirectly affect the formation of osteoclasts. **(B)** In M1 macrophages, miR-23a specifically inhibits A20, eventually reducing its own expression and promoting the expression of pro-inflammatory factors such as IL-1β, IL-6, IL12 and TNF-α, which promote M1 macrophage polarization and bone resorption. MiR-23a targeted JAK1 and STAT6, and then inhibits the polarization of M2 macrophages.

The function of miR-23a on osteoclasts is still being studied, but we assume that it promotes osteoclastogenesis in most cases, and its effect may depend on the bone microenvironment. It was found that upregulation of miR-23a in bone marrow-derived monocytes/macrophages can directly target and inhibit glycogen synthase kinase-3β (GSK3β) (110). As GSK3β inhibits osteoclast formation induced by RANKL (111), we suspect that miR-23a can promote osteoclastogenesis. The activation of JAK1 and STAT3 in osteocytes or T-cell by inflammation can provide RANKL to promote the formation of osteoclasts (112). JAK1 inhibitor has the effect of inhibiting bone loss (113), which inhibits osteoclastogenesis by repressing the expression of RANKL in osteoblasts (114). It is found that miR-23a can inactivate the JAK1/STAT3 signaling pathway (115), therefore, to some extent, miR-23a may prevent the excessive formation of osteoclasts. To determine if miR-23a can regulate osteoclastogenesis *via* altering the RANKL/OPG ratio or in other ways, more research is needed (**Figure 2A**).

### 3.2 MiR-23a and Macrophages

There is a double feedback loop between miR-23a and macrophage polarization (15). It was discovered that stimuli promoting the formation of M1 macrophages might restrict miR-23a expression, but M2 stimulation could boost it. In turn, miR-23a can regulate the polarization of macrophages or the balance between M1 and M2 types (15). The role of the NF-κB signaling in macrophage polarization has been explored, and it has been reported that the activation of this pathway can promote polarization of M1 macrophages while inhibition promotes polarization of M2 macrophages (116). In M1 macrophages, NF-κB binds to miR-23a promoter to inhibit its expression. On the contrary, miR-23a may directly target A20, the upstream inhibitor of NF-κB, to increase M1 macrophage polarization and the expression of M1 cytokines such as IL-1β, IL-6, TNF-α, and IL-12 (15, 117). IL-4/JAK1/STAT6 is an important pathway for the polarization of M2 macrophages. In M2 macrophages, STAT6 can promote the expression of miR-23a by binding to its promoter. However, miR-23a binds to the 3’ UTR of JAK1 or STAT6 to inhibit their expression and thus inhibit the polarization of M2 macrophages (15). In addition, macrophage-derived extracellular vesicles contain miR-23a-3p (118), which may be absorbed by members in the OB-OC-MΦ axis. Although the above research is not based on macrophages in bone microenvironment, based on the important role of macrophages in bone remodeling, it can be speculated that excessive miR-23a may result in the imbalance of the number of M1 and M2 macrophages, and affect other members in OB-OC-MΦ axis. In other words, overexpressed miR-23a may affect the polarization of macrophages, and eventually lead to the destruction of OB-OC-MΦ axis balance and the occurrence and progression of osteoporosis (**Figure 2B**).

### 3.3 MiR-23a and OB-OC-MΦ Axis

Members of the OB-OC-MΦ axis or other cells can secrete miR-23a through exosomes or extracellular vesicles, and miR-23a can be absorbed by osteoblasts, osteoclasts, macrophages and their precursors. Some factors can also directly cause miR-23a overexpression in these cells, resulting in OB-OC-MΦ axis imbalance. **Table 2** shows the sources, secretion modes, possible target genes and biological effects of miR-23a in bone microenvironment. What’s more, miR-23a may be a possible medium for the communication of members in the OB-OC-MΦ axis, which further enhances the destruction effect of miR-23a on this axis, leading to the occurrence and progression of osteoporosis. Overall, miR-23a plays a major role in regulating the OB-OC-MΦ axis, and targeted control of miR-23a is considered to be a potential strategy for therapeutic therapy of osteoporosis caused by OB-OC- MΦ axis imbalance.

**Table 2:** MiR-23a in bone microenvironment.

Sources	Target genes	Possible biological effects	Reference
Mast cell	Runx2 and SMAD1/5 in MSCs and osteoblasts	Inhibits osteoblast differentiation and mineralization; attenuates osteoblast maturation	(12)
Osteoclast	Runx2 in MSCs and osteoblasts	Suppresses osteoblast differentiation; Inhibits the activity of osteoblasts	(13)
Irradiation activated gingival fibroblast	CXCL12 in MSCs	Inhibits osteogenic differentiation of MSCs	(106, 107)
Macrophage	A20, JAK1 and STAT6 in macrophages	Increases M1 macrophage polarization; inhibits M2 macrophage polarization	(118)

## 4 MicroRNAs and Bone Diseases

### 4.1 Other microRNAs and OB-OC-MΦ Axis

MicroRNAs have developed into an important regulatory factor necessary to maintain bone homeostasis, which is of great significance to bone health and diseases (119). In addition to miR-23a, there are many microRNAs involved in the regulation of the OB-OC-MΦ axis, and there are synergistic or antagonistic effects among them.

Besides miR-23a, the extracellular vesicles released by neoplastic mast cells contain miR-30a, which can also inhibit Runx2 and SMAD1/5, and inhibit osteoblast differentiation and bone formation (12). MiR-17 and miR-31 are expressed in the process of osteogenic differentiation of adipose-derived stem cells (ASCs), and have inhibitory effects on osteogenesis mediated by BMP2 (101). This effect similar to miR-23a is related to miR-17 inhibiting BMP-2 expression and miR-31 inhibiting Osterix expression. What’s more, miR-103-3p directly targets methyltransferase-like 14 to inhibit osteoblast activity and bone formation (120).

In the process of OB-OC-MΦ axis regulation, there are also many microRNAs that have antagonistic effects with miR-23a. For instance, during the osteogenic differentiation of ASCs, the expression of endogenous miR-26a increases, which promotes osteoblast formation by inhibiting GSK3β (121). MiR-375 targets YAP1 (Yes-associated protein 1) to promote the osteogenic differentiation of ASCs (122); In MSCs, the overexpression of miR-130a can promote the osteogenic differentiation of MSCs and inhibit the formation of adipocytes, which is related to the fact that miR-130a directly binds the 3’UTR of Smad regulatory factors 2 and PPARγ (123). MiR-1246 activates the STAT3 signaling pathway and inhibits the NF-κB signaling pathway by targeting TERF2IP (telomeric repeat-binding factor 2-interacting protein) to induce M2 macrophage polarization (124). The inhibition of NF-κB signaling will reduce the production of pro-inflammatory cytokines, which further affects the OB-OC-MΦ axis.

It should not be ignored that there are complex positive and negative feedback loops between these miRNA and their targets (125). For example, miR-23a and A20/NF-κB, miR-23a and JAK1/STAT6, and miR-375 and YAP1 (15, 122). Many factors work together to maintain the balance of microRNAs and consequently the OB-OC-MΦ axis. When the expression leve of microRNAs is aberrant, the OB-OC-MΦ axis is destroyed inevitably, resulting in an imbalance between bone formation and bone absorption and, ultimately, increasing the incidence and progression of bone diseases such as osteoporosis.

### 4.2 MiR-23a and Other Bone Diseases

MiR-23a is implicated in the incidence and progression of various bone diseases, in addition to impacting osteoporosis by disrupting the balance of the OB-OC-MΦ axis. In rheumatoid arthritis, miR-23a can reduce bone loss and increase calcium retention by inhibiting the expression of LRP5, thus inhibiting the canonical WNT signaling pathway in cells (126). In osteosarcoma tissues and cells, miR-23a expression is decreased due to hypermethylation of promoter (127). The restoration of miR-23a expression can delay osteosarcoma cell proliferation, migration, and invasion. Connexin-43 is continually up-regulated during osteoblast differentiation, and its expression is also enhanced during human osteosarcoma cell differentiation. It has been revealed that miR-23a can target connexin-43 to prevent bone differentiation in osteosarcoma (128). Certainly, miR-23a can also inhibit the expression of other genes, such as Runx2 and CXCL12, to inhibit the progression of osteosarcoma (127).

MiR-23a may also affect bone diseases through synergistic or antagonistic effects of other microRNAs. For example, in osteonecrosis, miR-122-5p inhibits Sprouty2 and enhances the activity of receptor tyrosine kinase, thus promoting the proliferation and differentiation of osteoblasts (129). Similar to miR-23a, miR-137-3p targets Runx2 and CXCL12, and its silencing can promote bone formation and angiogenesis, thus preventing osteonecrosis. MiR-23a is an important member of the bone-related microRNA network. Targeted regulation of its expression has potential value for restoring the balance of OB-OC-MΦ axis and preventing and treating various bone diseases including osteoporosis.

## 5 Summary and Prospect

Osteoporosis is a disease that increases fracture risk due to decreased bone density. Osteoporosis will not only cost substantial medical care costs but also seriously reduce the life quality of patients and increase the mortality rate (130). Therefore, getting timely prevention and treatment is of great clinical and practical significance. In terms of mechanism, osteoporosis is mainly caused by bone resorption that is stronger than bone formation during bone remodeling. We first proposed the concept of the OB-OC-MΦ axis, pointed out the close connection between it and osteoporosis, and expounded the crucial role of miR-23a in osteoporosis induced by the imbalance of the OB-OC-MΦ axis.

Osteoblasts, osteoclasts, macrophages, and other members in the OB-OC-MΦ axis interact through direct contact, autocrine or paracrine to regulate bone homeostasis. Pathologically, the abnormal levels of inflammatory cytokines and hormones lead to the imbalance of the OB-OC-MΦ axis, enhanced bone resorption and inhibited bone formation, which promotes the occurrence and progression of osteoporosis. Similarly, the role of miR-23a in the occurrence and progression of osteoporosis is also realized through the OB-OC-MΦ axis. MiR-23a can inhibit bone formation by targeting the expression of genes encoding essential proteins in the process of osteoblast formation (101), among which miR-23a-Runx2, miR-23a-PGC-1α and miR23a-Wnt/β-catenin are the key ways. The high expression of miR-23a in macrophages promotes the polarization of M1 macrophages, stimulates the secretion of a variety of pro-inflammatory cytokines, and inhibits the polarization of M2 macrophages (14, 15). A variety of pro-inflammatory cytokines secreted by M1 macrophages, not only can inhibit the formation of osteoblasts, but also promote osteoclastogenesis (131). Simply put, the multiple effects of miR-23a on the OB-OC-MΦ axis make it easier for osteoporosis to occur when the level of miR-23a is high. Based on the multi-target and multi-pathway regulation effect of miR-23a on the OB-OC-MΦ axis, targeting miR-23a is expected to become an emerging potential and highly effective prevention and treatment strategy for osteoporosis.

Clinical trials targeting miRNAs are currently in full swing. As of 2018, more than 100 clinical trials have been initiated worldwide focusing on miRNAs for the treatment of psoriasis, cardiovascular disease, and cancer. In addition, miRNAs have been shown to be involved in various osteoclast-related diseases (132). Studies have shown that anti-miR-21 oligonucleotide inhibits the activity of miR-21 in aseptic loosening after arthroplasty, and the use of antagomir-21 in mouse tissues with particle-induced osteolysis improves osteolytic symptoms (133). In rheumatoid arthritis, regulating the expression of miRNAs such as miR-146a, miR-223, and miR-155 in peripheral blood mononuclear cells is a potential target for the treatment of rheumatoid arthritis (134–136).

Some clinical studies have been conducted to target miRNAs for the treatment of osteoporosis. Zoledronic acid (ZOL) inhibits the expression of RANKL by regulating miR-101-3p/RANKL, miR-302/PRKACB/RANKL and miR-145/SMAD3/RANKL signaling pathways. In HIV-positive subjects treated with tenofovir, down-regulation of RANKL expression by ZOL treatment alleviates osteoporosis (137). In patients with degenerative lumbar disease treated with posterior lumbar intervertebral fusion with cages, treatment with pluronic nanoparticles and oligosaccharide nanomedicine of alginate sodium reduces the occurrence of osteoporosis by regulating miR-155 (138). There are few clinical trials targeting miR-23a in bone disease, and only some studies have shown that icariin promotes osteogenic differentiation of bone marrow stem cells and improves osteonecrosis of the femoral head by reducing miR-23a-3p levels and regulating BMP-2/Smad5/Runx2 and WNT/β-catenin pathways (139). At present, there is no clinical trial targeting miR-23a for the treatment of osteoporosis. However, in view of the effect of miR-23a on inhibiting osteogenesis and promoting osteoclast- and macrophage-mediated inflammation, inhibiting the expression of miR-23a may become a crucial method for the treatment of osteoporosis. Further study of miR-23a may bring a new clinical horizon for the treatment of osteoporosis.

Detection of the expression of miR-23a in cells or tissues and targeted regulation of miR-23a provide potential strategies for the prevention and treatment of osteoporosis. However, the outcome of tailored modulation of miR-23a expression or cutting off relevant signal pathways in the prevention and treatment of osteoporosis, as well as the potential negative effects on the body, remains to be investigated. In general, the critical regulatory role of miR-23a in the OB-OC-MΦ axis provides a solid and sufficient theoretical basis for the multidisciplinary and multi-modal prevention and treatment of osteoporosis centered on miR-23a. With the in-depth study of miR-23a in osteoporosis, its clinical potential will be explored and applied.

## Author Contributions

T-LM and PZ wrote the original draft. T-LM, PZ, Z-RK and J-XC participated in writing and editing the review. PZ prepared the figures. Y-HH and JX edited the manuscript. All authors read and approved the final manuscript.

## Funding

This work was supported by the National Natural Science Foundation of China (Grant No. 81974339), the Science and Technology Plan Project of Hunan Province (Grant Nos. 2019JJ40499), and the National University Student Innovation Program (S2021105330596).

## Conflict of Interest

The authors declare that the research was conducted in the absence of any commercial or financial relationships that could be construed as a potential conflict of interest.

## Publisher’s Note

All claims expressed in this article are solely those of the authors and do not necessarily represent those of their affiliated organizations, or those of the publisher, the editors and the reviewers. Any product that may be evaluated in this article, or claim that may be made by its manufacturer, is not guaranteed or endorsed by the publisher.

## Abbreviations

ASC, adipose-derived stem cell; BMP, bone morphogenetic protein; CXCL12, C-X-C motif chemokine ligand 12; GC, glucocorticoid; gp130, glycoprotein 130; GSK3β, glycogen synthase kinase-3β; HSC, hematopoietic stem cell; IGF-1, insulin-like growth factor-1; JAK, Janus kinase; LRP5, low-density lipoprotein-receptor-related protein 5; M-CSF, macrophage colony-stimulating factor; miR-23a, microRNA-23a; miRNA, microRNA; MSC, mesenchymal stem cell; OB-OC-MΦ axis, osteoblast-osteoclast-macrophage axis; OPG, osteoprotegerin; OPN, osteopontin; OSM, oncostatin M; PPARγ2, peroxisome proliferator-activated receptor γ2; PTH, parathyroid hormone; RANK, receptor activator of nuclear factor κ-B; RANKL, receptor activator of nuclear factor κ-B ligand; Runx2, runt-related transcription factor 2; SMAD1, small mothers against decapentaplegic 1; STAB2, Stabilin 2; STAT3, signal transducer and activator of transcription 3; TGF-β, transforming growth factor β; TNF-α, tumor necrosis factor α; YAP1, Yes-associated protein 1.
